# Evaluation of Silver Nanoparticles Caffeic Acid Complex Compound as New Potential Therapeutic Agent against Cancer Incidence in Mice

**DOI:** 10.31557/APJCP.2021.22.10.3189

**Published:** 2021-10

**Authors:** Tahany Saad Abdelwahab, Rania Ellisy Abdelhamed, Eman Noaman Ali, Nahla Ahmed Mansour, Mohga Shafik Abdalla

**Affiliations:** 1 *Department of chemistry, Faculty of Science, Helwan University, Egypt. *; 2 *Department of Biochemistry, Radiation Biology Department, National Center for Radiation Research and Technology, Egypt. *; 3 *Department of Organic Chemistry, Petrochemical Department, Egyptian Petroleum Research Institute, Egypt. *

**Keywords:** Apoptosis, conjugated caffeic and folic acid, tumor, flow cytometric analysis, radiation, silver nanoparticles

## Abstract

**Objective:**

The present work was designed to study the effect of new conjugated caffeic and folic acid with silver nanoparticles with definite molecular size applied with and without gamma radiation exposure, as an antitumor agent against experimentally induced Ehrlich tumor and attempted to identify their potential molecular mechanisms of action throughout determination of anti-tumor activities using MTT cytotoxic assay against two human carcinoma cell lines in vitro, such as apoptosis analysis by flow cytometry through caspase-8, caspase-3 and TNF determination in vivo.

**Materials and Methods:**

Adult female albino mice were used and divided into five groups. Animals were sacrificed and the following parameters were estimated, glutathione (GSH), glutathione peroxidase (GPx), superoxide dismutase (SOD) in blood in addition to caspase8, caspase 3 and tumor necrosis factor (TNF) of tumor tissue, liver and kidney function also measured in plasma. The tumor specimens were processed for histopathological examination.

**Results:**

Nano-silver folate caffeic (NSFC) complex compound treatment resulted in growth inhibition in Ehrlich solid tumor, Hep-G2, and MCF-7 cells (IC50 0.062 mg, 7.70 µM, and 14.50 µM, respectively). Flow cytometric analysis revealed that (NSFC) with radiation IR had apoptotic effects at caspases 8 (Mean±SD) (49.4±14), caspase3 (39.97±9.75), and TNF (40.1±3.4) more than any other groups. Those disturbances were found to be associated with a kinetic induction of apoptosis and showed modulation of the antioxidant system {glutathione (GSH), glutathione peroxidase (GPx) and superoxide dismutase (SOD) which were 60.70±0.80, 26.73±0.80, 39.52±0.58 respectively}at the group which took (NSFC+IR), besides its high percentage of necrotic cells by histopathological studies.

**In conclusion,:**

the present study showed that the treatment of (NSFC) exhibits very efficient oncolytic activity in delaying tumor growth in mice bearing Ehrlich Solid Carcinoma (ESC) and the mechanisms underlying the inhibitory effect of the present compound involve both an apoptotic effect against Hep-G2 and MCF-7 cells and modulation of antioxidant system.

## Introduction

Cancer is a group of diseases that spread quickly by the uncontrolled growth of abnormal cells and lead to death (Small Jr et al., 2017). The propagation of cancer is continuing to increase, although efforts to relieve risk factors in recent decades. 

Chemotherapy and radiotherapy are known for worthy adverse effects, kill only tumor cell but they discovered, they kill both tumor and health cells (Navya et al., 2019). 

Many side effects develop as a result of indefinite targeting anticancer factors and destitute drug delivery of those factors cannot adapt the appropriate outcome in most cases. Various methods are applied for cancer treatment, each of which has some side effects and critical restrictions. The uncontrolled propagation of cancer cells where apoptosis has greatly voided. Feel necessity for very complicated means of treatment (Jabir et al., 2016). 

The main objective of cancer therapeutics becomes organizing the drug to differentiate the cancerous cells and the normal cells to decrease their proliferation. Chemotherapy causes serious side effects. It declines to select the targeted cancerous cells without affecting the normal body cells (Al Mamun et al., 2018). 

It is essential to join chemotherapy with other cancer treatments by design a drug delivery system, to overthrown these restrictions and obtain better cancer therapeutic efficiency, prevailing tendency and challenges in cancer therapy using nanomaterials (Zhao et al., 2018).

For cancer treatment, critical strides have been made in the direction of applying engineered nanomaterials with high specificity, sensitivity, and efficacy. Nanotechnology has the potential to avoid the disadvantages of traditional therapeutic formulations (You and Henneberg, 2018).

Biochemical events end in characteristic cell changes and death. In multicellular organisms, apoptosis is a type of programmed cell death that is done. Cell shrinkage, nuclear fragmentation, chromatin condensation, Blebbing, chromosomal DNA fragmentation, and global mRNA decay are the changes that have occurred. Apoptosis is originated through intrinsic and extrinsic pathways; it is a highly controlled process. Two pathways activate cell death by mobilizing caspases, which are protease enzymes that play a role in the ER transduction of apoptotic signals and divide proteins (Kollek, 2016). caspases have two types: initiator caspases, including caspase 2, 8, 9,10,11,12, and effectors caspases included caspase 3, 6, 7. The initiator caspases were activated by both pathways, which then induced executioner caspases, which degrading proteins randomly to kill the cell (Nagata, 2018). Apoptotic cell death can induce by tumor necrosis factor (TNF) which is an endogenous pyrogen and has an important role within the immune cell regulation and can inhibit tumorigenesis and viral replication (Kordulewska et al., 2018). 

The nanosilver particles can interact with membrane proteins and induced signaling pathways, led to inhibition of cell proliferation by causing mitochondrial dysfunction, generation of reactive oxygen species (ROS), and damage to proteins and nucleic acids inside the cell (Akter et al., 2018).

Under chemotherapy and radiation treatments of cancer, caffeic acid (CA) has an important role in protecting organs in animal models. Also, it has been shown to sensitize cancer cells by inhibiting pathways that lead to more resistance. It is potentially a cancer therapeutic agent due to shows very little or no toxic side effects. Treatment with CA may protect patients from radiation therapy or chemotherapy, not only suppress tumor growth in patients (Akyol et al., 2012). CA triggered TRAIL-induced apoptosis by regulating mitogen-activated protein kinases (Kim et al., 2013). The present findings revealed that a potential mechanism by which CA could enhance the effects of TRAIL through CHOP-mediated DR5 expression, suggesting that CA/TRAIL-mediated apoptosis originates from DR5 (Gieffers et al., 2013). Tumor necrosis factor-related apoptosis-inducing ligand (TRAIL) is a promising anti-cancer agent because of its capability to induce apoptosis in various cancer cell types, but with negligible cytotoxic effects in normal cells (Mongkolsapaya et al., 1998). TRAIL mediates apoptosis by binding to two transmembrane agonistic cell surface receptors, death receptor 4 (DR4) and DR5 (Jung et al., 2015). Subsequent interaction of DR4 or DR5 with the adaptor protein, Fas-associated death domain (FADD), leads to the activation and recruitment of caspase-8 (Woo et al., 2013). Ultimately, activation of caspase-8 activates executioner caspase-3 that mediates apoptotic cell death CHOP is known as an apoptotic inducer through endoplasmic reticulum (ER) stress and that depletion of CHOP prevents apoptosis against various cancer drugs (Tabas and Ron, 2011). However, recently accumulated data confirmed that ER stress accelerated CHOP expression, leading to the promotion of the expression of ER stress-mediated apoptotic molecules, such as DR5 (Yamaguchi and Wang, 2004).

Folic acid is one of the water-soluble vitamins B9, also known as folate (the naturally occurring form) and exists in both cancerous cells and healthy cells due to the folic acid receptors on the cell surfaces, the number of these receptors depended on the cell functions. Due to the vast requirement of cancerous cells for folic acid tend to overexpress the folic acid receptors (Mariadoss et al., 2020). It has been advised that folate may prevent cancer, as it is involved in the synthesis, repair, and functioning of DNA (Silva et al., 2017). The folic acid receptor makes an appropriate targeting agent due to its relative overexpression level in the cancerous cell, low expression in normal tissues (Angelopoulou et al., 2019). The powerful aspect of folic acid is its possible to conjugate with nanosilver. when these nanosilver particles accumulated at cancerous cells (Chauhan et al., 2017). 

The present study was designed to evaluate the efficiency of nanosilver functionalized with folate as a targeted compound with caffeic acid as a promising anticancer complex with or without radiation therapy. we were founded that Nano-Silver Folate Caffeic (NSFC) complex compound inhibits the transformation of normal cells to cancer cells as well as suppressing the proliferation of several human cancer cell lines and have also been shown to sensitize cancer cells to chemotherapeutic drugs and radiation treatments by inhibiting pathways that lead to treatment resistance as well as protecting important organs in animal models (Gan et al., 2018).

## Materials and Methods

Tested compound: (NSFC) complex compound, silver nitrate, folic acid, caffeic acid, the starting materials were purchased from Sigma-Aldrich Chemical Co. (St. Louis, Mo, USA) and dimethyl sulfoxide, triethylamine, dicyclohexyl carbodiimide, N-hydroxysuccinimide (NHS) from EIMC united pharmaceuticals, Egypt. 

Cell cultures (in-vitro): Besides to Ehrleish solid tumor, two cancer cell lines, human hepatocellular carcinoma cell line (Hep-G2) and breast carcinoma cell line (MCF-7) (VACSERA, Egypt). The cells were cultured in RPMI-1640 medium supplemented with 10% fetal bovine serum (FBS) and 100 units/ml penicillin and 2.00 mg/ml streptomycin. The cell cultures were maintained at 37°C in a humidified 5% CO_2_ atmosphere.

Experimental Animal (in-vivo): Female Swiss albino mice weighing 25-30 g obtained from (the animal house of the Ain Shams University, Egypt) were used in this study. The animals were divided into five experimental groups (15 mice each) as follows: group (1) untreated control group, group (2) mice bearing Ehrlich solid carcinoma (ESC) ((2.5*106) viable EAC cells in 1ml saline and mouse was injected in right thigh of the lower limb by 0.1 ml ), group (3) mice bearing ESC exposed to ionizing radiation as fractionated doses (2Gy / week for 3 weeks), group (4) mice bearing ESC treated with NSFC intraperitoneal at a dose of ( 0.42 mg of AgNPs/ ml of NSFC) for each time through 45 days group (5) mice bearing ESC treated with NSFC as in group 3 and exposed to irradiation as in group 2. Mice were allowed to become acclimatized to laboratory conditions for one week before the experiment and were housed at a constant temperature (24±2^o^C) with alternating 12 h light and dark cycles and fed standard laboratory food and water. The study protocol was approved by the Institutional Animal Ethics Committee and all the animal experiments were carried out under the guidelines of the Committee for Control and Supervision of Experiments on Animals (VACSERA), Egypt. The ethics number is HU.IACUC.BioChem./MA-1119/05.

Transplantation of solid tumor: Solid tumors were produced by intramuscular inoculation with 0.20 ml of Ehrlich Ascites Carcinoma (EAC), which contained (2.5*106) viable EAC cells, in the right thigh of the lower limb of each mouse. Mice with a palpable solid tumor mass (100 mm³) that developed within 10 days after inoculation were used in the study. The change in tumor volume (TV) was measured at different time intervals during the experimental period (days 10, 13, 16, 20, 23, 27, 30, 34, 38, 41, and 44) using a Vermeer caliper and calculated by the following formula according to (Mohamed et al., 2015). Tumor volume (mm³) = 0.52 AB², where A is the minor axis and B is the major axis.

*Preparation of our new tested compound*

A-Conjugation of caffeic and folic acid: The method was carried out according to that of (Gao et al., 2012), with some modifications using a reported method with a minor modification (Chan et al., 2007). Folic acid N-hydroxysuccinimide active ester (FA-NHS) was synthesized through the following method: a solution of anhydrous dimethyl sulfoxide and triethylamine (200:1) was stirred for 10 min. 0.5 g folic acid was added gradually to the above mixture and continuously stirred in the dark overnight. Folic acid was mixed with 0.2 g dicyclohexylcarbodiimide and 0.2 g NHS and stirred for a further 24 h. The by-product dicyclohexylurea (DCU) was removed by filtration. DMSO and triethylamine were evaporated under a vacuum. Then the conjugation of caffeic-folic acid was carried out through dissolving of vacuum-dried FA-NHS into the mixture of DMSO and trimethylamine (2:1) and 0.2 g caffeic acid was added to the mixture and stirred overnight (Khademi et al. 2018), to obtain the conjugated caffeic-folic acid solution.

*B- Surface modification of AgNPs with caffeic -folic acid conjugate*

The functional groups in the conjugated caffeic-folic acid solution play a role in the reduction of silver nitrate during the formation of AgNPs. Firstly, 0.17 gm of AgNO_3_ was dissolved in 250 ml of water, then it was stirred for about 20 min at room temperature and dropwise from our conjugated solution was added until all silver was precipitated which mean silver coated by active folate-caffeic acid in nanoformulation (NSFC) compound (Lin et al., 2017).

*Characterization techniques*

UV-visible absorption of the NSFC complex compound was measured by using a UV-Vis spectrophotometer (JascoV-750-Japan) at room temperature conditions. Transmission electron microscopy (TEM) was recorded using a JEM-2100F (JEOL), Japan, at an acceleration voltage of 200 kV and used to investigate the size of the NSFC complex compound. A few drops of NSFC solution were diluted into 1 ml of water, and the resulting solution was placed onto a carbon-coated copper grid and allowed to evaporate. The spectrometer model type Perkin-Elmer 1650 FTIR spectrophotometer in the range of 4,000 – 400 cm^-1^ was used to identify the infrared spectroscopy (FTIR) of a sample, where the KBr technique was used. X-ray diffraction (XRD) measurements were performed on Philip’s X-ray diffractometer PW1390 with Ni-filtered CuKα radiation at generator voltage of 40 kV and wavelength of 0.154 nm at room temperature. The diffraction angle, 2θ, was scanned at a rate of 2 deg/min (Al-Mahweet, 2020). JCPDS file No. of AgNO_3 _is 04-0783.

*The MTT cell viability assay (in-vitro)*

The cytotoxic effect of (NSFC) complex compound against Hep-G2 and MCF-7 cells was measured using the MTT cell viability assay. MTT (3-[4, 5-dimethyl thiazole-2-yl]-2,5 diphenyl tetrazolium bromide), which is based on the cleavage of the tetrazolium salt by mitochondrial dehydrogenizes in viable cells. Cells were treated for 24 h with various concentrations of NSFC before being submitted to the MTT assay. The relative cell viability was expressed as the mean percentage of viable cells comparing to DMSO-treated cells, and the half-maximal growth inhibitory concentration (IC_50_) was calculated by the trend line equation (Kumar et al., 2018).

*(In-vivo) study “experimental animal”*

*Samples collection*

Blood sampling: At the end of the experiment, Directly, after animals were anesthetized, blood was collected from the heart by heparinized syringes, transferred to 2 sets of heparinized tubes, allow standing for 15 min at room temperature, then a set of tubes were centrifuged at 3500 r.p.m. for 15 min using (Heraeus Sepatech centrifuge) to separate plasma for determination of parameters of liver and kidney functions. The other set of blood collection served in the determination of glutathione reduced form (GSH), glutathione peroxidase (GSH-Px), and superoxide dismutase (SOD). Tissue sampling: As quickly as possible-after animals were anesthesia and sacrificed, the tumor was dissected out and kept in 10% formalin for histopathological examination till the time of the investigation.

Flow cytometry assay: The flow cytometry assay was performed at (Genetic unit, Children hospital, Mansoura University, Egypt). A FACS Caliber flow cytometry (BD Company, USA) was used to calculate the percentages of cells marker surface by using the Intuitive Software, BD Accuri C6 Plus software. Files were exported in FCS 3.1 format for seamless data import into flow cytometry analysis programs such as FCS Express™ software and Flow Jo™ software. Samples were run in triplicate and each experiment was repeated three times. Doublets and higher aggregates were excluded by gating as described in the results section. Measurements were performed at rates of 50–450 events per second at the lowest or medium flow rate, with 10,000 events (excluding doublets) being recorded per sample (Herzenberg et al., 2002).

Tissue preparation and fixation: tumor tissue samples previously were thawed in cold PBS/ EDTA. Small pieces (about 3 x 3 x 3 mm) of such thawed specimens were mechanically dispersed, sequentially using 100 μm and 35 μm nylon cell strainers (BD Falcon #352360 and #352235). 5 ml, -20°C, cold 80% ethanol; were added dropwise under constant, gentle vortexing. Samples were incubated for 30 mins on ice and subsequently overnight at -20°C before being subjected to staining. Cell suspensions were removed from -20°C storage and allowed to adapt to room temperature (RT) for ~10 mins. After centrifugation (310 xg; RT; 6 mins), pellets were resuspended in 5 ml PBS/EDTA (RT), incubated for 5–30 min at RT, and pelleted again (310 xg; RT; 6 mins). Cell sediments were suspended in an appropriate volume (0.5–2 ml) of staining solution (PBS without EDTA, containing (specific antibodies to every marker). Samples were then incubated for 30 mins in a 37°C water bath in the dark, cooled down to room temperature, and subjected to flow cytometry (Heinlein et al., 2010). 

Caspase-3 assay: After preparation and fixation of cells as mentioned above, followed by the anti-caspase-3 antibody (Purified Rabbit Anti- Active Caspase-3 Clone C92-605 (RUO), Concentration 0.5 mg/ml, Catalog No.559565, BD Pharmingen™), for 30 mins at 22ºC. Acquisition of > 5,000 total events was collected using a 20mW Argon ion laser (488nm) and 525/30 bandpass filter. Gating strategy events were collected with the forward and side light-scatter characteristics (Kong et al., 2019).

Caspase-8 assay: After preparation and fixation of cells as mentioned above, followed by the anti-caspase-8 antibody (Purified Rabbit Anti- Active Caspase-8 Clone 3-1-9 (RUO), Concentration 0.5 mg/ml, Catalog No. 551242, BD Pharmingen™), for 30 mins at 22ºC. Acquisition of > 5,000 total events was collected using a 20mW Argon ion laser (488nm) and 525/30 bandpass filter. Gating strategy events were collected with the forward and side light-scatter characteristics (Kong et al., 2019).

*Tumor Necrosis Factor-alpha assay*

After preparation and fixation of cells as mentioned above, followed by the anti-TNF-a antibody (Purified Rabbit Anti- Active TNF-a clone MP6-XT22 (RUO), concentration 0.2 mg/ml, catalog No. 561063, BD Pharmingen™), for 30 mins at 22ºC. Acquisition of > 5,000 total events was collected using a 20mW Argon ion laser (488nm) and 525/30 bandpass filter (Kong et al., 2019).

*Glutathione “GSH” determination by the colorimetric method*

The method was carried out according to that of (Beutler et al., 1963). It was based on spectrophotometric measurement of the yellow color of 2-nitro-5-thiobenzoic acid which was produced as one product.

*Glutathione peroxidase “GSH-Px” determination by the colorimetric method*

The method was carried out according to that of (Paglia and Valentine, 1967). The method is a linked enzyme reaction in which the oxidized glutathione formed by the action of H_2_O_2_ and GSH-Px is converted back to its reduced form in the presence of glutathione reductase (GSH-R) and NADPH. The amount of residual GSH left after exposure to enzyme activity for a fixed time was measured calorimetrically. GSH-Px activity is proportional to the amount of GSH that is consumed in the reaction.

*Superoxide dismutase “SOD” determination by the colorimetric method*

The method was carried out according to that of (Minami Yoshikawa, 1978). Superoxide dismutase (SOD) catalyzes the dismutation of the superoxide radical (O^2-^) into hydrogen peroxide (H_2_O_2_) and elemental oxygen (O_2_). Superoxide ions, generated from the auto-oxidation of pyrogallol, converted the chloride nitro blue tetrazolium (NBT) to NBT-diformazan which absorbs light at 550 nm. SOD reduces the superoxide ion concentration and thereby lowers the rate of NBT-diformazan formation. The extent of reduction in the appearance of NBT-diformazan was a measure of SOD activity present in samples. 

*Histopathological examination*

The method was carried out according to that of (Bancroft et al., 1996). After sacrificing, liver, kidney, and tumors (taken from the thigh muscle of mice with implanted Ehrlich tumor cells in different groups) were removed and placed into 10% formalin for 24 h and decalcification occurred on formic acid. Washing was done by tap water then serial dilutions of alcohol (methanol, ethanol, and absolute ethanol) were used for dehydration. Specimens were cleared in xylene and were embedded in paraffin at 56 degrees in a hot air oven for 24 h. Paraffin bees wax tissue blocks were prepared for sectioning at 4 microns thickness by slidge microtome. The obtained tissue sections were collected on glass slides deparaffinized and then stained by hematoxylin and eosin “H&E” stain. Then examination was done through the light electric microscope.

*Statistical Analysis*

Data were analyzed using one-way analysis of variance (ANOVA) using the statistical package for social science (SPSS) program (Miller, 2017). Numerical data were expressed as mean ± S.D, minimum and maximum. Qualitative data were expressed as frequency and percentage. 

## Results

*The tested compound*

**Figure 1:**
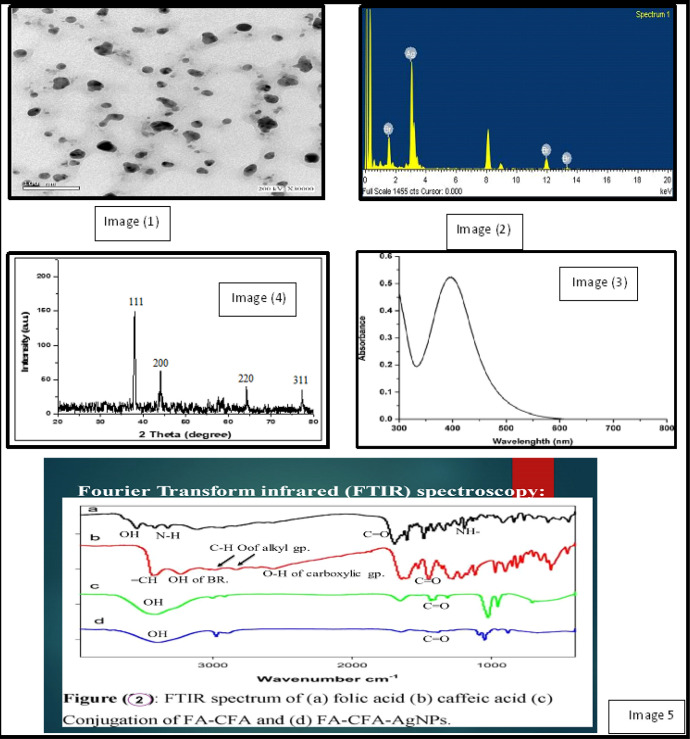
Image (1), TEM images of synthesized Ag NPs; Image (2), EDX spectrum of synthesized Ag NPs; Image (3), XRD patterns of synthesized AgNPs (JCPDS file No. of AgNO3 is 04-0783); Image (4), UV-visible absorption of silver nanoparticles; Image (5), FTIR spectrum of (a) folic acid (b) Caffeic acid (c) Conjugation of FA-CFA and (d) FA-CFA-AgNPs

A-Particle size determination: The analysis was carried out by using a transmission electron microscope (TEM) for the AgNPs synthesized by folic-caffeic acid conjugate solution. It showed that AgNPs are spherical and not in physical contact with each other. This observation may suggest that AgNPs have a silver core, and folic-caffeic acid conjugate surrounds this core. It was demonstrated that, diameter particles in the 10–20 nm range. Figure 1, image (1).

B- Energy Dispersive investigation of X-Ray: The prepared sample was confirmed using the Energy Dispersive investigation of the X-Ray beam (EDX) system, (Jeol sort JSM-T200, Japan). Figure 1, image (2).

C- X-ray diffraction (XRD): After washing, the particles are collected by ultracentrifugation and analyzed by XRD to further verify that they were Ag NPs. As shown in Figure 1, image (3a), revealed that the synthesized NPs are found with diffraction peaks. Also, Figure 1, image (3b) represented the EDX profile of Ag NPs, showing strong signals related to elemental silver confirming AgNPs formation.

D- Fourier transform infrared (FTIR) spectroscopy: FTIR is a technique to understand the intermolecular interaction by strong and specific non-covalent bonds. Exact information about the functional groups involved in the interaction process would be given by FTIR spectra at Table (1). As shown in Figure 1, image (4a), the bands for pure folic acid. Figure 1, image (4b) showed the beaks of caffeic acid. The conjugation of caffeic and folic acids is shown in Figure 1, image (4c). While, the FTIR spectrum of FA-CA-AgNPs Figure 1, image (4d), showed the same bands with a slight shift to lower wave number.

E- UV Spectroscopy: The absorption spectrum of the silver nanoparticles (after washing) is presented in Figure 1, image (5). The sample presents the characteristic surface plasm of silver nanoparticles. Ag nanoparticles existing in a narrow band with a maximum at 403 nm in the UV-visible absorption spectrum further confirmed the Ag+ to Ag0 conversion.

Using this formula, the mean crystallite length for Ag NPs was 1.27 nm. This is Agree with the TEM photo values. Also, (image 2) represented the EDX profile of Ag NPs, showing strong signals related to elemental silver confirming AgNPs formation. the AgNPs synthesized by Folic-Caffeic acid conjugate showed that the AgNPs are spherical and not in physical contact with each other. This observation may suggest that AgNPs have a silver core, and Folic-Caffeic acid conjugate surrounds this core. Although TEM images show diameter particles in the 10–20 nm range. The presence of plasm on resonance absorption peak at 403 nm in the UV-visible absorption spectrum confirmed of the Ag nanoparticles due to reduction by caffeic-folic complex.

*The MTT cell viability assay (in-vitro)*

**Figure 2:**
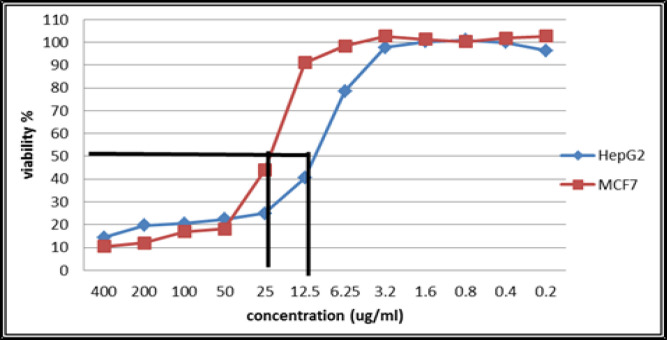
Cell viability of Hep-G2 and MCF-7 cell lines with NSFC at different concentrations

Anti-tumor activity against human hepatocellular carcinoma cell line (Hep-G2) and breast carcinoma cell line (MCF-7):

Cytotoxicity was measured and expressed as the survival fraction compared with untreated control cells. The possible anti-proliferative effect of (NSFC) complex compound was studied on the growth of our tumor cell lines after incubation for 24 h. The treatment of Hep-G2 and MCF-7 cells with NSFC complex compound dramatically inhibited the cell growth in a dose-dependent manner as shown at Table 2 and Figure 2, with IC_50_ values of 7.70 µM 14.50 µM respectively.

*(In-vivo) study “experimental animal”*

**Figure 3:**
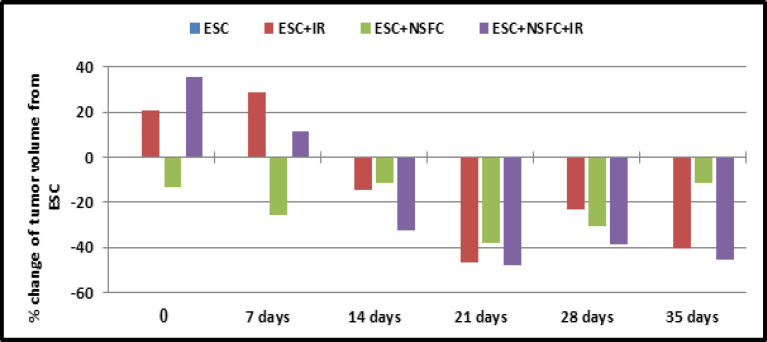
Percentage of Change of the Tumor Volume Comparing with ESC Group/ Weak

In the present study, the effect of tumor inoculation in mice with or without different types of treatments was evaluated and compared either with the control group, or mice bearing tumor group as shown at Table 3 and Figure 3. 

*Flow Cytometry assay*

**Figure 4:**
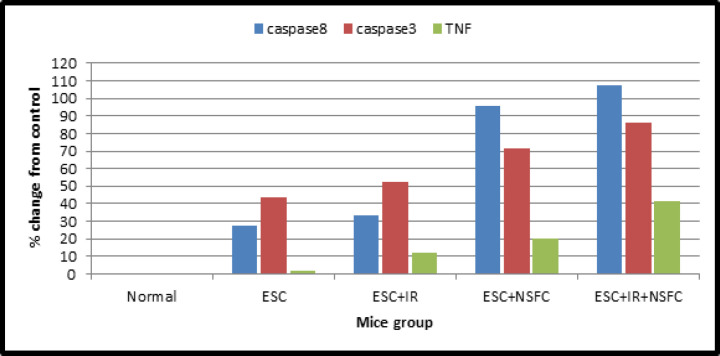
Percentage Change of Caspase-8, -3, and TNF Contents from Control

As shown in the data that have been summarized in Table 4 and Figure 4, the caspase-8, -3 and TNF content was measured in tumor of ESC mice and the different treated groups.

**Figure 5:**
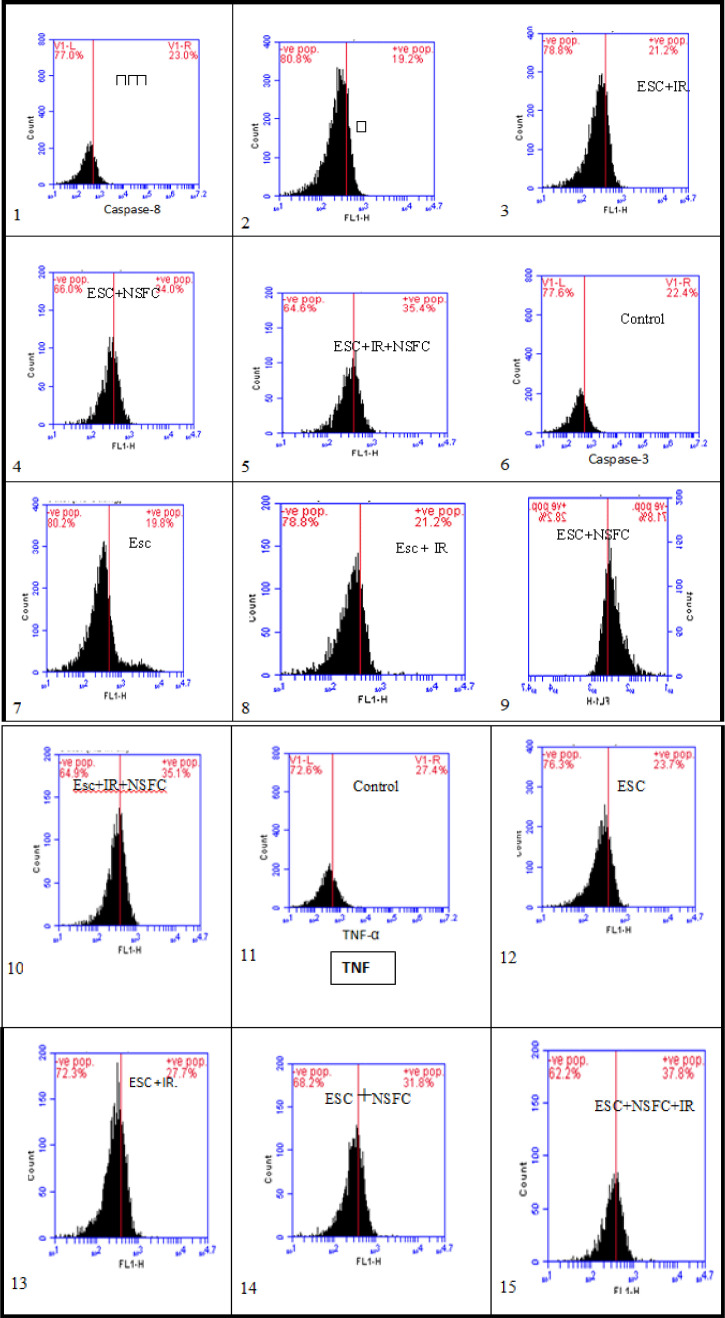
Image (1:5) represented caspase-8 [1(Control), 2(ESC), 3(ESC+IR), 4(ESC+NSFC) and 5(ESC+NSFC+IR)], image (6:10) represented caspase-3 [6(Control), 7(ESC), 8(ESC+IR), 9(ESC+NSFC) and 10(ESC+NSFC+IR)], image (11:15) represented TNF [11(Control), 12(ESC), 13(ESC+IR), 14(ESC+NSFC) and 15(ESC+NSFC+IR)].

Figure 5 also represented the % cell viability in each group and for each parameter (caspase-8, -3 and TNF).

*Glutathione content (GSH), Glutathione peroxidase “GSH-Px” and Superoxide dismutase “SOD”*

**Figure 6:**
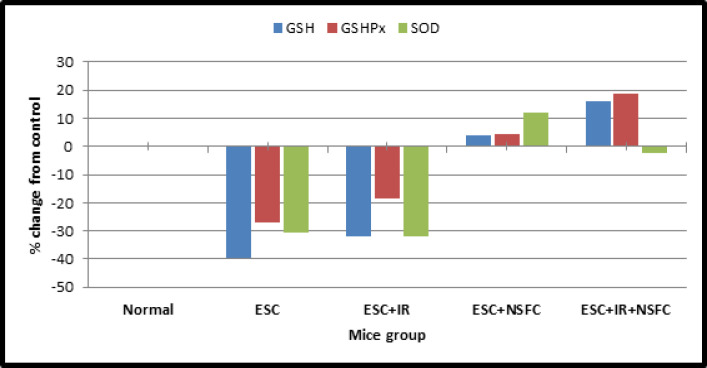
The Percentage Change of Antioxidants Parameters Contents from Control

As shown in the data that have been summarized in Table (5) and Figure (6), the glutathione (GSH), glutathione peroxidase “GSH-Px”, and superoxide dismutase “SOD” contents were measured in the blood of control mice and the different treated groups.

*Histopathological examination*

**Figure 7:**
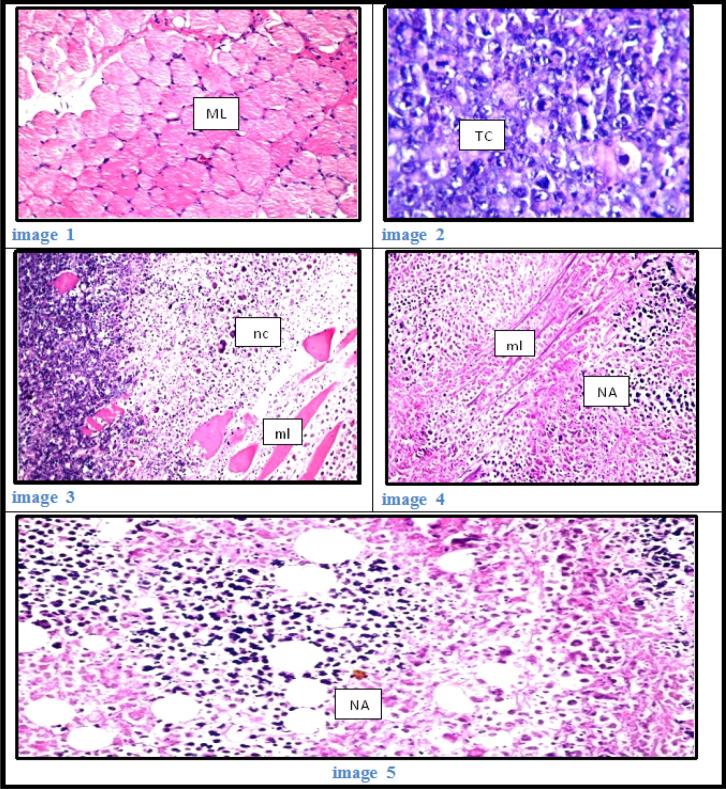
Image 1: Thigh muscle of mice in group 5 showing normal histological structure of striated muscle bundles (ML). (H&E, x40) Image 2: Thigh muscle of mice in group 2 showing a massive number of inter neoplastic Ehrlich tumor cells (TC) with criteria of malignancy (H&E, x40). Image 3: Thigh muscle of mice in group 3 showing the necrosis and apoptosis (nc) of the tumor cells, muscles bundles (ml) (H&E, x40). Image 4: Thigh muscle of mice in group 4 showing the necrosis (NA) of the tumor cells as well as the muscle bundles (ml) (H&E, x40). Image 5: Thigh muscle of mice in group 5 showing the necrosis and apoptosis (NA) in the tumor cells (H&E, x40).

As shown at Figure 7:

Control group (1), was showed no histopathological alteration, and normal histological structure of the striated muscle bundles.

Group (2) mice bearing Ehrlich solid carcinoma (ESC), Massive numbers of the Ehrlich tumor cells were occupied the muscle bundles associated with inflammatory cells infiltration, degeneration and atrophy in the bundles and was showed criteria of malignancy as nuclear hyperchromachia, mitosis as well as cellular pleomorphism.

Group (3) mice bearing ESC with radiation (ESC+IR), a wide area of the tumor cells showed necrosis and apoptosis with degeneration and necrosis in the muscle bundles

Group (4) mice bearing ESC treated with NSFC (ESC+NSFC), the Ehrlich tumor cells showed diffuse necrosis and apoptosis associated with necrosis also in the muscle bundles.

Group (5) mice bearing ESC treated with NSFC with radiation (ESC+NSFC+IR), there were wide.

**Table 1:** FT-IR assignment of Nano-Silver Folate Caffeic Complex Compound

Assigned (cm^-1^)	Vibrational mode	Functional group
3545	O-H stretching band	O-H of glutamic acid
3416	N-H stretching band	N-H of glutamic acid
1694	C=O stretching vibration	(C=O) carboxylic group
1606	NH- vibration	NH-
1484	stretching vibration	Phenyl & Pterin ring
1640	Carbonyl stretching vibration	Amide group (-CONH2)
3431	Stretching beak	Caffeic acid
2985	=CH stretching	Alkyl group CH2
2832	C-H stretching	Alkyl group CH
2562	O-H stretching	(COOH) carboxylic group
3231	O-H stretching	O-H benzene ring
1644	C=O stretching	(COOH) carboxylic group
3416	OH stretching	(OH) Hydroxyl group
1654	C=O stretching	(-COOH) carboxylic group

**Table 2:** Inhibition Concentration 50 (IC_50_) of NSFC Compound against Hep-G2 and MCF-7 Cell Lines

NSFC Concentration (µM)	Cell Viability (Hep-G2)	Cell Viability (MCF-7)
400	14.38	10.51
200	19.72	11.91
100	20.56	16.88
50	22.47	18.19
25	24.97	43.99
12.5	40.49	91.01
6.25	78.54	98.49
3.2	97.78	102.61
1.6	100.21	101.38
0.8	100.9	100.36
0.4	99.93	101.81
0.2	96.32	102.75
IC_50_ (µM)	7.70	14.50

**Table 3:** Mean±SD of Mice Tumor Volume Every Week and Percentage of Change from Tumor Cases

Duration of tumor volume measurements (mm^3) Mean±SD												
Animal group	7 days after tumor inoculation without treatment		14 days from tumor inoculation and 7 days of treatment		21 days from tumor inoculation and 14 days of treatment		28 days from tumor inoculation and 21 days of treatment		35 days from tumor inoculation and 28 days of treatment		42 days from tumor inoculation and 35 days of treatment	
	Mean ±SD	Sign.	Mean ±SD	Sign.	Mean±SD	Sign.	Mean±SD	Sign.	Mean±SD	Sign.	Mean±SD	Sign.
ESC	0.53±0.36	NS^e,c,d^	1.55±0.46	NS^c,d,e^	5.09±2.9	NS^c,d^ 0.061^e^	10.39±3.6	0.001^c,e^ 0.007^d^	13.46±5.0	NSC 0.042^d^ 0.011^e^	13.6±6.09	0.012^c^ 0.005^e^ NS^d^
ESC+ IR	0.64±0.53	NS^e,b,d^	2.00±10.06	NS^b,e^ 0.005^d^	4.35±1.3	NS^b,d,e^	5.56±2.9	0.001^b^ NS^d,e^	10.43±5.3	NS^b,d,e^	8.09±2.18	0.012^b^ 0.065^d^ NS^e^
ESC+NSFC	0.46±0.21	NS^e,c,b^	1.15±0.2	NS^b^ 0.005^c^ 0.043^e^	4.50±2.0	NS^b,c,e^	6.43±3.2	0.007^b^ NS^c,e^	9.34±4.1	NS^c,e^ 0.042^b^	12.05±6.10	0.065^c^ 0.032^e^ NS^b^
ESC+NSFC+IR	0.72±0.42	NS^b,c,d^	1.73±0.45	NS^b,c^ 0.043^d^	3.45±0.7	NS^c,d^ 0.061^b^	5.40±2.6	0.001^b^ NS^d,c^	8.24±2.2	NS^c,d^ 0.011^b^	7.42±2.74	0.005^b^ 0.032^d^ NS^c^

ESC, mice bearing Ehrlich solid carcinoma; ESC+IR, mice bearing tumor exposed to ionizing radiation; ESC+NSFC+IR, mice bearing tumor treated by nano-silver folate caffeic complex compound; Mean±SD , mean of tumor volume of 10 mice (gm); sign., (signification); signification calculated by One Way ANOVA at 0.05, b signification from ESC, c signification from ESC+IR, d signification from ESC+NSFC, e signification from ESC+NSFC+IR and NS nonsignificant.

**Table 4:** Molecular Parameters of Mice Groups as Mean±SD and Percentage of Change from Tumor Cases

Molecular parameters						
Mice group	Caspase-8		Caspase-3		TNF	
	Mean±SD	Sign.	Mean±SD	Sign.	Mean±SD	Sign.
Control	23.83±0.73	NS^b,c^ 0.017^e^ 0.028^d^	21.5±0.95	0.090^d^ 0.048^e^ NS^b,c^	28.36±0.91	NS^b,c,d^ 0.057^e^
ESC	30.45±11.25	NS^c,d,a^ 0.093^e^	30.87±11.3	NS^c,d,e,a^	28.8±8.8	NS^c,d,e,a^
ESC+IR	31.8±10.6	NS^e,b,d,a^	32.73±11.6	NS^e,b,d,a^	31.8±7.9	NS^e,b,d,a^
ESC+NSFC	46.6±12.6	NS^b,c,e^ 0.028^a^	36.93±12.2	0.090^a^ NS^b,c,e^	34.1±8.2	NS^b,c,e,a^
ESC+NSFC+IR	49.4±14	NS^c,d^ 0.093^b^ 0.017^a^	39.97±9.75	NS^b,c,d^ 0.048^a^	40.1±3.4	0.057^a^ NS^b,c,d^

ESC, mice bearing Ehrlich Solid Carcinoma; ESC+IR, mice bearing tumor exposed to Ionizing Radiation; ESC+NSFC+IR, mice bearing tumor treated by nano-silver folate caffeic complex compound; Mean±SD, mean of tumor volume of 10 mice (gm); Sign, signification which calculated by One Way ANOVA at 0.05; ^a^, signification from control; ^b^, signification from ESC; ^c^, signification from ESC+IR; ^d^, signification from ESC+NSFC; ^e^, signification from ESC+NSFC+IR and NS nonsignificant.

**Table 5:** The Antioxidants Parameters of Mice Groups as Mean±SD and % of Change from the Normal Group

Antioxidants parameters						
Mice group	GSH		GSH-Px		SOD	
	Mean±SD	Sign.	Mean±SD	Sign.	Mean±SD	Sign.
Control	52.22±1.34	0.000^b,c,e^ 0.062^d^	22.49±0.79	0.000^b,c,e^ 0.094^d^	40.53±0.85	0.000^b,c,e^ NS^d^
ESC	31.62±1.65	0.000^a,d,e^ 0.003^c^	16.46±0.55	0.000^a,d,e^ 0.004^c^	28.10±1.19	0.000^a,d,e^ NSc
ESC+IR	35.47±1.12	0.000^a,d,e^ 0.003^b^	18.33±0.37	0.000^a,d,e^ 0.004^b^	27.55±0.61	0.000^a,d,e^ NS^b^
ESC+NSFC	54.30±0.95	0.000^b,c,e^ 0.062^a^	23.43±0.47	0.000^b,c,e^ 0.094^a^	45.42±0.96	0.000^b,c,e,a^
ESC+NSFC+IR	60.70±0.80	0.000^a,b,c,d^	26.73±0.80	0.000^a,b,c,d^	39.52±0.58	NS^a^ 0.000^b,c,d^

ESC, mice bearing Ehrlich Solid Carcinoma; ESC+IR, mice bearing tumor exposed to ionizing radiation; ESC+NSFC+IR, mice bearing tumor treated by nano-silver folate caffeic; Mean±SD, mean of tumor volume of 10 mice (gm); Sign, signification, signification calculated by One Way ANOVA at 0.05; ^a^, signification from control; ^b^, signification from ESC; ^c^, signification from ESC+IR; ^d^, signification from ESC+NSFC; ^e^, signification from ESC+NSFC+IR and NS nonsignificant.

## Discussion

Chemotherapy drugs are cytotoxic to tumor cells, but their lack of specificity results in a variety of side effects. There’s an urgent need for more novel and efficacious therapeutic agents and techniques for the treatment of breast cancer one among the foremost formidable female malignancies. The off-target effects of such drugs will be improved through the utilization of nanoparticles (NPs). Administered nanoparticles enhanced accumulation in tumor tissue near the blood vessels, promoting both anticancer drug permeability and tumor retention. Several nanocarriers are now approved for clinical use in an exceedingly range of cancer therapies, and plenty of novel formulations are within the later stages of clinical trials (Jin et al., 2020). Within the present study new formula of nanoparticles, the complex compound was synthesized. We focus specifically on a model of breast cancer transplanted in animals and discuss the longer term of NPs as potential treatment options in these areas.

The silver nanoparticle has been tested to has a significant role in the field of oncology, as its use as a nanoprobes for tumor detection and imaging, and as an inhibitor for tumor progress and suppression of angiogenesis (Ebrahimzadeh et al., 2020).

In-vivo application, mice bearing tumor, treated by novel complex compound (NSFC)with or without radiation (IR) exposure revealed depression in tumor volume compared with mice bearing tumor with none treatment (ESC), but the group that treated with (NSFC+IR) together revealed more pronounced within the depression of tumor volume.

The lifetime of mice during the experiment period wasn’t suffering from radiation or novel compound treatment. Also, there was no toxicity feature noticed at either bearing tumor or healthy animals exposed to NSFC and/ or radiation exposure along the amount of the experiment.

Present investigation strongly suggests that (NSFC) complex compound, additionally to its high potential antitumor activity in-vitro, has also a radical scavenging effect in-vivo by encountering free radicals after tumor cells inoculation which revealed marked amelioration within the depleted GSH content and scavenger enzymes, (GSH Px and SOD) activities within the blood of tumor-bearing mice, treated by novel complex drug (NSFC) with or without radiation exposure (IR). As shown within the present study, there’s a correlation between tumor presences and over-production of (ROS) which has been liable for the deleterious pathogenesis resulted from the presence and growth of Ehrlich ascites carcinoma. As mentioned by Yadav et al., (2019), impairment of mitochondrial respiration was evident from reduced oxygen consumption detected in tumor cell lines.

NSFC complex compound can be able to induce apoptosis but its mechanism as an antioxidant isn’t fully understood. Therefore, the initiation and execution of the apoptotic cell death program induced by ROS act as signaling molecules. The cellular antioxidants such as glutathione act as reversible redox modifiers of the function of an enzyme in addition to managing ROS levels; this proved the action of ROS as signaling molecules (Zou et al., 2017).

The apoptosis process plays a significant role in tissue homeostasis and development, so apoptotic regulation linked with human diseases propagation. Therefore, this process may be a definite class of programmed cell death. The death cell is regulated by the interaction between the two pathways of survival and apoptosis. The cell fate can determine by the changes between the survival and apoptosis pathways. the involving proteins inside the survival pathway are reduced in the case of cancer, while proteins within the apoptotic pathway are appeared (Codispoti et al., 2019).

In the Present study determination of caspase8, caspase3 and TNF recorded a rise in their levels detected in mice bearing tumor treated by novel complex compound (NSFC) with or without IR compared with control healthy mice group or untreated bearing tumor mice (ESC) group that indicated the activation of apoptosis through caspase cascade. In agreement with our finding, previous reports showed that radiation exposure activates caspase8 which results in induced apoptosis in cancer cells as mentioned by Safavi et al., (2018). Although their results and therefore the data of others initially suggested that caspase-8 activation in response to ionizing radiation or cytostatic drugs implied the involvement of death receptor pathways.

Since radiation also induced apoptosis in caspase-8 cancer cells, tested whether caspase-3 remains activated in those cells. Irradiation with 6 Gy delivered as fractionated doses (2 Gy /week for 3 weeks) induced activation of caspase-3. The addition of novel complex compounds with radiation exposure enhanced caspase 8 and caspase 3 levels. These results were under those of Liu et al., (2018).

The anti-carcinogenic action of CA is mainly associated with its antioxidant (Sidoryk et al., 2018) and pro-oxidant capacities (Zhang et al., 2008), and this is attributed to its chemical structure. Firstly, the presence of free phenolic hydroxyls (ortho-dihydroxyl) makes it possible to reduce the enthalpy of OH-bond dissociation and increase the transfer rate of H atoms for peroxyl radicals, as well as their number and position on the phenyl ring (catechol group). Also, the presence of a double bond in the carbon chain (the unsaturated side chain 2,3 double bond) increases the stability of the phenolic radical (Touaibia et al., 2011), These chemical factors associated with the CA molecule enable the elimination of free radicals, preventing the production of ROS as well as the induction of DNA oxidation of cancer cells present in various types of cancer (Silva et al., 2014)

CA has a great potential for reducing metals due to its structural chemical characteristics; the compound is susceptible to auto-oxidation and also oxidation caused by another biological agent. The molecular structure of CA, containing a catechol group with an α, β-unsaturated carboxylic acid chain, is responsible for its interaction with various types of oxidizing radicals (Damasceno et al., 2017).

Histopathological examination within the present study demonstrated that, regression of the dimensions of neoplastic nodules at the location of tumor inoculation after treatment with the novel promising compound NSFC. Moreover, there was a significant decrease in tumor volume observed in NSFC treated group compared with the untreated one. Histopathological examination of muscle sections of tumor-bearing untreated group ESC founded massive numbers of the Ehrlich tumor cells were occupied the muscle bundles related to inflammatory cells infiltration, degeneration, and atrophy within the bundles and showed criteria of malignancy as nuclear hyperchromachia, mitosis also as cellular pleomorphism. But in mice bearing tumors and treated by unique complex compound with or without radiation exposure (ESC+NSFC+IR) group, there have been wide areas of necrosis and apoptosis within the tumor cells also as within the muscle bundles.

It is acknowledged that the defects in caspase cascade activation cause tumor progression and metastasis while its activation by novel complex compound (NSFC) sensitizing tumor cells to (IR) leads to induction of apoptosis, reduction in tumor size, and control of cancer development. The amplification in an expression of caspase 3 protein after radiation exposure induces cell death through the apoptotic pathway.

In conclusion, from the aforementioned results, based on our findings, we can conclude that the nanosilver folate caffeic complex compound has good cytotoxic activity against the human liver carcinoma cell line and breast carcinoma cell line tested in the thesis. Flow cytometric apoptosis analysis has revealed that (NSFC) has an apoptotic effect at caspases 8, 3, and TNF more than any compound of them alone. While (NSFC) compound showed modulation of an antioxidant system than any compound of them alone also besides its high percentage of necrotic cells and apoptotic cells by histopathological studies. The present study proved that, (NSFC) alone or with (IR) is better than treated with IR alone or treated by any compound of them alone in cancer treatment with more future researches. And finally, it is worth mentioning once again that the effect of the applied novel promising synthetic compound NSFC needs more experimental trails to explore different mechanisms could this novel compound uses to fight the tumor, also developed and applied this new class of cancer drugs as radiopharmaceuticals, which deliver radiation therapy directly and specifically to cancer cells. 

## Author Contribution Statement

M.S.A The main supervisor of the search and corresponding author. T.S, E.N and M.S.A conceived and designed research. T.S conducted experiments, wrote the manuscript. R.E. and T.S analyzed data. E.N, T.S and N.A.M new reagents or analytical tools. All authors read and approved the manuscript and all data were generated in-house and that no paper mill was used. Funding: this work was supported by Helwan University with self-funding.
